# Immunity to Respiratory Infection Is Reinforced Through Early Proliferation of Lymphoid T_RM_ Cells and Prompt Arrival of Effector CD8 T Cells in the Lungs

**DOI:** 10.3389/fimmu.2019.01370

**Published:** 2019-06-14

**Authors:** Jenny E. Suarez-Ramirez, Karthik Chandiran, Stefan Brocke, Linda S. Cauley

**Affiliations:** Department of Immunology, University of Connecticut Health Center, Farmington, CT, United States

**Keywords:** immune regulation, respiratory infection, cytotoxic T cell, viral immunity, immunological “memory”

## Abstract

Cross-protection between serologically distinct strains of influenza A virus (IAV) is mediated by memory CD8 T cells that recognize epitopes from conserved viral proteins. Early viral control begins with activation of tissue-resident memory CD8 T cells (T_RM_) cells at the site of viral replication. These CD8 T cells do not act in isolation, as protection against disseminated infection is reinforced by multiple waves of effector cells (T_EFF_) that enter the lungs with different kinetics. To define how a protective CTL response evolves, we compared the functional properties of antiviral CD8 T cells in the respiratory tract and local lymphoid tissues. When analyzed 30 dpi, large numbers of antiviral CD8 T cells in the lungs and mediastinal lymph nodes (MLNs) expressed canonical markers of T_RM_ cells (CD69 and/or CD103). The check point inhibitor PD-1 was also highly expressed on NP-specific CD8 T cells in the lungs, while the ratios of CD8 T cells expressing CD69 and CD103 varied according to antigen specificity. We next used *in vitro* experiments to identify conditions that induce a canonical T_RM_ phenotype and found that that naïve and newly activated CD8 T cells maintain CD103 expression during culture with transforming growth factor-beta (TGFβ), while central memory CD8 T cells (T_CM_) do not express CD103 under similar conditions. *In vivo* experiments showed that the distribution of antiviral CTLs in the MLN changed when immune mice were treated with reagents that block interactions with PD-L1. Importantly, the lymphoid T_RM_ cells were poised for early proliferation upon reinfection with a different strain of IAV and defenses in the lungs were augmented by a transient increase in numbers of T_EFF_ cells at the site of infection. As the interval between infections increased, lymphoid T_RM_ cells were replaced with T_CM_ cells which proliferated with delayed kinetics and contributed to an exaggerated inflammatory response in the lungs.

## Introduction

The 1918 influenza pandemic caused more deaths in the period of a single year than any other emerging infectious disease (1). Intermittent infections with new strains of avian influenza A virus (IAV) raise concerns that another global pandemic could begin at any time (2, 3). Efforts to protect public health include regular vaccination with inactivated viral products. Because the antibodies do not bind viruses with modified surface proteins, these vaccines provide little protection against infection with new strains. Frequent vaccine failures emphasize the need for broadly protective vaccines that target multiple different serotypes (4). As models predict that dependence on a single vaccine could increase the severity of future pandemics (5), preparations for mass vaccination should encompass different methods of immunization. Since a local route of vaccine delivery is required to populate the lungs with antiviral memory CD8 T cells (6), vectored vaccines may be the best method for inducing broad immunity in the respiratory tract (7).

Cytotoxic T lymphocytes (CTLs) enhance immunity by destroying host cells that support viral replication (8–10). Responses to new strains of IAV develop slowly, while naïve CD8 T cells undergo clonal expansion in the local lymph nodes. Several days pass before effector CD8 T cells (T_EFF_) enter the lungs and destroy host cells that support viral replication (8–10). During the delay, replicating virus spreads to the lower respiratory tract, where T cell-derived cytokines contribute to defuse alveolar damage (11, 12). We have previously shown that less damage occurs when viral dissemination is impeded by tissue-resident memory CD8 T cells (T_RM_) in the airways (6). The role of T_RM_ cells in immunity was discovered after MHCI tetramers were used to quantify CTLs in the lungs during the recovery phase of infection. Investigators found that the numbers of memory CD8 T cells in the circulation did not change while protective immunity declined (13, 14). Importantly, some anti-viral CTLs expressed CD69 in the lungs and gradually disappeared as protective immunity declined. The presence of these activated CTLs prompted us to explore how long viral peptides were presented to CD8 T cells during the recovery phase of infection. We found that the mediastinal lymph node (MLN) contained residual viral peptides until at least 2 months after intranasal (i.n.) inoculation (15). Importantly, the remaining peptides induced abortive proliferative responses from naïve CD8 T cells, while T_CM_ cells show no signs of activation (16). After completing several rounds of cell-division, the responding cells displayed a partially-activated phenotype as indicated by increased CD44, CD11a, and CD69 expression.

We used parabiosis experiments to explore how antiviral memory CD8 T cells survey the lungs during recovery from infection (15, 17). These experiments showed that some CD8 T cells left the circulation during acute viral infection and remained lodged in the walls of the airways after infectious virus had been eliminated (15, 18). A majority of the resident cells displayed a canonical T_RM_ phenotype, exemplified by stable CD69 and/or CD103 expression. In addition, more than 80% of antiviral CD8 T cells in the MLNs were non-circulating (host-derived) cells that expressed CD69 and/or CD103 (15, 17), including some cells that expressed the immune checkpoint inhibitor programmed death-1 (PD-1). For parabiosis experiments, we used mice that were housed in specific pathogen free (SPF) facilities. As expected, very few CD8 T cells expressed CD69 or PD-1 before IAV infection. In contrast, both markers were widely expressed on CD8 T cells in lymph nodes recovered from human cadavers and out-bred mice that had been exposed to diverse environmental pathogens, indicating a response to microbial products (19, 20).

Although the contributions of mucosal T_RM_ cells to antiviral immunity are widely recognized, the functional properties of lymphoid T_RM_ cells remain poorly defined (21). Here 5-bromo-2′-deoxyuridine (BrdU) has been used to analyze the proliferative responses of antiviral memory CD8 T cells in the lungs and local lymphoid tissues after heterosubtypic challenge. By altering the length of time between primary and secondary IAV infection, we show that defenses in the lungs of immune mice are reinforced by early proliferation by T_RM_ cells in the lung-draining lymph nodes and prompt arrival of T_EFF_ cells at the site of viral replication.

Multiple receptors control access to the circulation, including CD69 and CD103 (αeβ7 integrin) which is expressed on some subsets of CD8 T cells during stimulation with TGFβ. Studies have shown formation of pulmonary T_RM_ cells requires local exposure to antigen and/or TGFβ (17, 22, 23), but we have limited knowledge of the signals that are involved in maintenance of these specialized cell populations. For the current study, we compared the surface antigens on T_RM_ cells that recognize three different viral epitopes and found that phenotypes of lymphoid T_RM_ cells varied according to antigen-specificity. T_RM_ cells that were specific for an epitope encoded in the nucleoprotein (NP) gene expressed surface markers that were consistent with a response to persisting viral peptides (i.e., CD69 and/or PD-1, without CD103). PD-1 was highly expressed on CD8 T cells in the lungs. In contrast, CTLs that were specific for an epitope encoded in the acid polymerase (PA) gene included larger percentages of cells that expressed CD103 in combination with CD69, while PD-1 was largely absent. A two-step culture system has been used to explore how circulating CD8 T cells respond to stimulation with TGFβ. Our data indicate that responses to this cytokine are influenced by the timing and quantity (or context) of antigen exposure in local tissues.

## Materials and Methods

### Mice and Reagents

OTI mice (24) were bred and housed at UCONN Health, in accordance with institutional guidelines. C57BL/6 mice were purchased from Charles River. Frozen MHCI molecules (NP_366−374_/D^b^, PA_324−333_/D^b^) were supplied by the NIH tetramer facility (Emory University, Vaccine Center at Yerkes, Atlanta, GA) and (OVA_257−264_/K^b^) MBL International corporation. Tetramers were made at UCONN Health. Virus stocks were grown in fertilized chicken eggs (Charles River), titered and stored as described previously (15). Between 8 and 20 weeks after birth, anesthetized mice were infected intranasally with 2 ×10^3^ PFU WSN-OVA_I_ (H1N1) (25). For secondary infections, mice received 5 ×10^3^ PFU X31-OVA (H3N2) (26). Blocking antibodies to PD-L1 (B7H1) and isotype control were purchased from Bio-Xcell (West Lebanon, NH.). Mice received 250 μg of blocking antibody (100 μl saline) given twice by i.p. injection. Mice were infected (i.v.) with 5,000 CFU recombinant *Listeria monocytogenes* expressing chicken ovalbumin (LM-OVA) (27). Experiments were performed in accordance with guidelines and protocols approved by the University of Connecticut Health Center Institutional Animal Care and Use Committee (IACUC).

### Adoptive Cell Transfer and Sample Preparation for Flow Cytometry

Naïve CD8 T cells were isolated from spleens and pLN of CD45.1^+^ OTI mice using Mojosort isolation kits (Biolegend). OTI cells were labeled with CFSE according to directions from the manufacturer (Molecular Probes). Mice received 3 ×10^5^ OTI cells by I.V. injection. For flow analysis, chopped lung tissue was digested using 150 U/ml collagenase (Life Technologies, Rockville, MD, USA) in RPMI containing 1 mM MgCl_2_, 1 mM CaCl_2_, 5% FBS and incubated at 37°C for 90 min. Non-adherent cells were enriched on 44/67% Percoll gradients and spun at 400 *g* for 20 min. Washed lymphocytes were incubated with antibodies that block Fc-receptors for 15 min, then stained with anti-CD8 and MHCI tetramers for 1 h at room temperature. Antigen-experienced CTLs were identified using high CD44 and CD11a expression. NP-specific CTLs were phenotyped using Tetramer-PE, CD103-AF647, CD69-PerCP, and PD-1 FITC. OVA and PA-specific CTLs were phenotyped using Tetramer-APC, CD103-FITC, CD69-PerCP, and PD1-PE. BrdU was administered by i.p. injection 4 h before cell analysis. BrdU incorporation was measured by intracellular staining, according to instructions from the manufacturer (BD Biosciences). To visualize cells that were close to X or Y axis, scales on some contour plots were adjusted using the bi-exponential function in Flowjo^®^ software.

### Cell Culture

Naïve CD8 T cells were stimulated with plate-bound anti-CD3/CD28 and rIL-2 (20 U/ml) in 24 well plates. Cultures were supplemented with SB-431542 (10 μM) or vehicle (0.1% DMSO) (28). Other wells were exposed to exogenous TGFβ (10 ng/ml). Cells were suspended in RPMI containing FBS, L-glutamine, β-mercapthoethanol, sodium pyruvate, Hepes, and antibiotics. At 48 h, CTLs were transferred to new wells and stimulated with activated TGFβ (10 ng/ml) and rIL-2 for an additional 48 h (no antigen).

### Confocal Microscopy

MLNs were fixed in 4% PFA/PBS for 60 min at 4°C and cut into thick sections (350 microns) using a vibratome. Sections were pre-incubated with antibodies to block Fc-receptors (15 min at 4°C) and stained with biotin-conjugated antibodies to CD11c (eBioscience) diluted in 2% FBS/PBS solution. After extensive washing, sections of fixed MLNs were stained overnight at 4°C with streptavidin-PE antibody (Life Technologies), Pacific blue-conjugated anti-CD31, Alexa Flour 488- conjugated anti-CD45.1 (BioLegend, San Diego, CA, USA), eF660-conjugated LYVE-1, and B cells were detected with V500-conjugated anti-B220 (BD Biosciences). After extensive washing, stained tissues were mounted on slides using Shandon Immu-Mount (Thermo Electron, Pittsburgh, PA, USA). Images were recorded using a Zeiss LSM880 confocal microscope with an inverted Axio Observer. Fluorescence was detected using: an argon laser for emissions at 458, 488, and 514 nm; a diode laser for emissions at 405 and 440 nm; a diode-pumped solid-state laser for emissions at 561 nm; and a HeNe laser for emissions at 633 nm. Images were analyzed using the colocalization function in Imaris suite software (Bitplane, South Windsor, CT, USA).

### Histology

Lungs were fixed in 4% PFA/PBS at 4°C for 24–48 h. After washing, lungs were stored in 70% ethanol until processing. Hematoxylin and eosin (H&E) staining was performed by the Histology Core at the UCONN Health. Images were takes at 5X and 20X normal magnification.

### Statistical Analysis

Statistical significance was determined using an unpaired two-tailed Student *t*-test. Horizontal lines indicate comparisons between samples, with *p* values from groups of 5/6 mice. NS, *P* > 0.05; ^*^P < 0.05; ^**^P < 0.01; ^***^P < 0.001; ^****^P < 0.0001.

## Results

### Lymphoid T_RM_ Cells Are Receptive to Signals That Promote Lodgment in Peripheral Tissues

**Figure 1:**
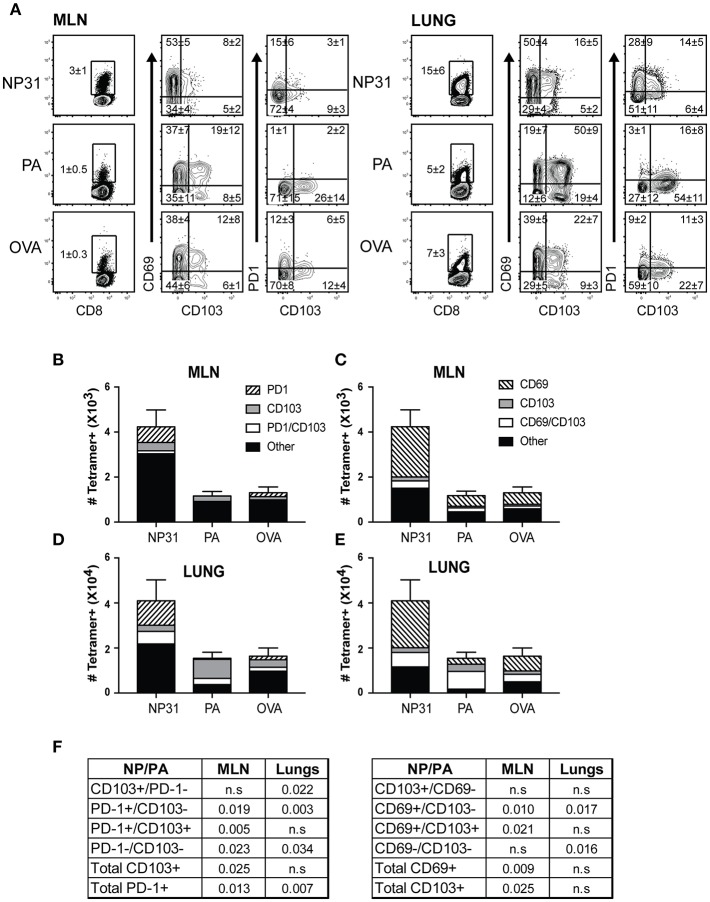
The phenotypes of T_RM_ cells vary according to antigen-specificity. Antiviral CTLs were recovered from the lungs and MLN 35 dpi with WSN-OVA_I_. Antigen experienced CTLs were identified using high CD11a and CD44 expression. **(A)** Contour plots show antigen-experienced CTLs analyzed with MHCI tetramers. The tetramer^+^ CTLs were analyzed for CD103, CD69, and PD-1 expression. Percentages of cells in each quadrant are means ± SD (*n* = 5/group). Gates were set using non-CD8 T cells. **(B–E)** Bar graphs show total numbers of tetramer^+^ CTLs, plotted using means ± SD (*n* = 5/group). Shading shows **(B,D)** ratios of cells expressing CD103 and/or PD-1, **(C,E)** ratios of cells expressing CD103 and/or CD69. **(F)** The numbers of CTLs in each quadrant were compared for the NP and PA epitopes. *P*-values were calculated using Student's *t*-tests.

To examine the phenotypes of antiviral CD8 T cells during recovery from IAV infection, C57BL/6 mice were infected with a recombinant virus (WSN-OVA_I_) encoding the SIINFEKL peptide (25). Virus-specific CD8 T cells were analyzed 30 dpi, using MHCI tetramers containing peptides encoded by the nucleoprotein (NP_336−374_/D^b^), acid polymerase (PA_224−233_/D^b^), and ovalbumin genes (OVA_257−264_/K^b^). The lungs and MLNs both contained antiviral CD8 T cells that expressed canonical markers of T_RM_ cells (Figure 1A). NP-specific CD8 T cells were the dominant subset at both locations (Figures 1B–E). CD69 was expressed on large percentages of antiviral CD8 T cells identified with all three tetramers. In contrast, the percentages of virus-specific CD8 T cells that expressed CD103 and/or PD-1 varied according to antigen-specificity (Figure 1A). A majority of NP-specific CD8 T cells lacked CD103, while PD-1 was highly expressed in lungs. Conversely, only small percentages of PA-specific CTLs expressed PD-1 and CD103 was highly expressed in the lungs. The OVA-specific CTLs displayed an intermediate phenotype. Gates for analyses were set using non-CD8 T cells.

**Figure 2:**
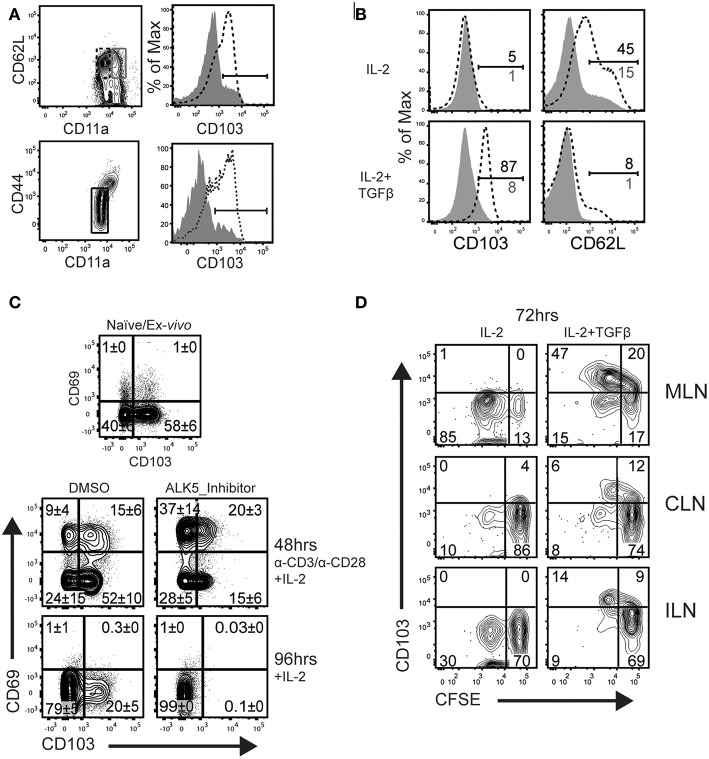
T_CM_ do not upregulate CD103 during culture with TGFβ. **(A)** C57BL/6 mice were infected systemically (i.v.) with LM-OVA. CD62L-positive CD8 T cells in the spleen were analyzed 32 dpi. The contour plots indicate gates that were used for analyses. Histograms (top row) show CD103 expression on naïve CD8 T cells, while T_CM_ cells do not express CD103 (gray shading). Lower panels show CD103 expression naïve OTI cells from the spleens of uninfected mice (bottom row, dashed line). Naïve OTI cells do not express CD103 after ablation of the TGFβ receptor (bottom row, gray shading). **(B)** Naïve OTI cells were transferred to B6 mice 48 h before infection with LM-OVA. After 3 months, T_CM_ cells were sorted from the spleens using CD45.1 expression. Purified CD8 T cells (naïve and T_CM_) were stimulated with plate-bound anti-CD3/CD28 and rIL-2 (20 U/ml) for 48 h. Live cells were transferred to new wells (no antigen) and cultured for 48 h with rIL-2 plus/minus TGFβ (10 ng/ml). **(C)** Naïve OTI cells were cultured with plate-bound anti-CD3/CD28 and rIL-2. In addition, some wells were supplemented with SB-431542 (10 μM) to avoid stimulation with serum-derived TGFβ. After 48 h, activated OTI cells were analyzed for CD103/CD69 expression (top panels). Additional cells were cultured for an additional 48 h with rIL-2 and no antigen stimulation (lower panels). **(D)** Naïve OTI cells were labeled with CFSE-dye and transferred to mice that were previously (30 d) infected with WSN-OVA_I_. The MLNs were recovered 5 days after transfer and lymphocytes were cultured for 72 h with rIL-2 plus/minus TGFβ. Three experiments gave similar results.

Tonic signaling from the TGFβ receptor reinforces expression of αeβ7 integrin (CD103) on T_RM_ cells (17, 29). We next investigated whether naïve and/or circulating memory CD8 T cells express CD103 *in vivo*. We previously found that T_CM_ cells develop with delayed kinetics after IAV infection, due to the influence of persisting viral peptides. To avoid this complication, C57BL/6 mice were infected with recombinant *L. monocytogenes* encoding the chicken ovalbumin gene (LM-OVA) (27). After 32 days, CD8 T cells were recovered from the spleens and analyzed for CD103 expression (Figure 2A). After gating CD62L^+^ cells, naïve and central memory CD8 T cells (T_CM_) were distinguished using CD44 and CD11a (top row). The overlaid histograms show that CD103 expression on naïve CD8 T cells, but not T_CM_ cells. Similarly, CD103 was expressed on naïve CD8 T cells from uninfected mice and was absent when the TGFβ receptor was not expressed (bottom row).

We used a transfer model to create a homogeneous supply of OVA-specific T_CM_ cells for *in vitro* experiments. Naïve CD8 T cells were recovered from the peripheral lymph nodes of OTI mice which express a transgenic receptor that is specific for SIINFEKL peptide presented in the context of H-2K^b^ (OVA_257−264_/K^b^) (24). Enriched OTI cells were transferred to C57BL/6 mice 48 h before infection (i.v.) with LM-OVA. After 3 months, T_CM_ cells (CD62L^high^/CD11a^high^) were sorted from the spleens using CD45.1 expression. A two-step culture system was used to compare the phenotypes of naïve and T_CM_ cells after stimulation with TGFβ (Figure 2B). To induce cell proliferation, purified OTI cells (naïve and T_CM_) were stimulated with plate-bound anti-CD3/CD28 and recombinant IL-2 (20 U/ml). After 48 h, live cells were transferred to clean wells and cultured for an additional 48 h in fresh media, supplemented with rIL-2, plus/minus TGFβ (10 ng/ml). The naïve OTI cells down regulated CD103 during antigen stimulation and expression did not return during extended culture with rIL-2. Importantly, CD103 was re-expressed when the cultures were supplemented with TGFβ. Conversely, T_CM_ cells lacked CD103 expression when cultured under similar conditions.

Respiratory infection promotes formation of mucosal T_RM_ cells which express CD103 in combination with CD69. CD69 is expressed on CD8 T cells soon after antigen stimulation (or exposure to selected cytokines), and quickly disappears when the stimulus is removed (30). Here, cultured CTLs were used to identify conditions that induce the canonical T_RM_ phenotype of dual CD69 and CD103 expression (Figure 2C). Naïve OTI cells were stimulated with anti-CD3/CD28 and rIL-2 as previously described. Fetal bovine serum contains small quantities of TGFβ. To prevent stimulation with this cytokine, replicate wells were supplemented with an inhibitor (SB-431542) that prevents phosphorylation of TGFβ receptor I (ALK5) (31), or the vehicle (DMSO) control. The first samples were analyzed 48 h after antigen stimulation, when approximately 20% of OTI cells expressed CD69 in combination with CD103 (top row). The percentages of CD69^+^ cells increased when the ALK5 inhibitor was present, while CD103 was partially down-regulated. Higher percentages of CD69^+^ CD8 T cells indicate that the inhibitor prevented suppression from TGFβ during early T cell activation (32). Other OTI cells were stimulated with antigen for 48 h and transferred to new wells containing fresh medium with rIL-2, for an additional 48 h without antigen (lower panels). Approximately 20% of OTI cells maintained CD103 expression during extended culture with rIL-2, while both markers (CD103 and CD69) were down regulated when the cultures contained the ALK5 inhibitor (SB-431542) (Figure 2C). These data confirmed CD103 expression on newly activated CD8 T cells is reinforced by stimulation with TGFβ.

We previously used transfer experiments to determine how long viral peptides were presented to CTLs in the tissues of IAV infected mice (15). To detect persisting OVA peptides, naïve OTI cells were labeled with CFSE-dye and transferred to mice that had previously been infected with WSN-OVA_I_. The donor cells were analyzed 5 days after transfer, when reduced intensity of the CFSE-dye showed that some OTI cells proliferated *in vivo*. Here, a similar protocol was used to determine whether CD8 T cells remain receptive to TGFβ after completing several rounds of cell division in the MLN. C57BL/6 mice were infected with WSN-OVA_I_ and CFSE-labeled OTI cells (CD11a^low^/CD44^low^) were transferred 30 dpi. After 5 days, lymphocytes were recovered from the MLNs and cultured for 48 h with rIL-2 (20 U/ml), plus/minus TGFβ (10 ng/ml). Diluted CFSE-dye showed that some OTI cells divided in the MLN between 30 and 35 dpi and maintained CD103 expression during culture with TGFβ (Figure 2D). Together, these studies show that naïve CD8 T cells respond to “late” antigen stimulation in the MLN and remain receptive to environmental cues that encourage lodgment in peripheral tissues. Importantly, T_CM_ did not respond to late antigen presentation (16), or express CD103 when cultured with TGFβ.

### The Distribution of Lymphoid T_RM_ Cells Changes During Therapeutic Blockade of PD-1L

PD-1 is a costimulatory molecule that delivers negative-signals to T cells during interactions with APCs. Newly activated CTLs transiently express PD-1 during antigen stimulation, while stable expression has been linked to chronic antigen exposure and suboptimal T_EFF_ function (33, 34). During chronic infections, many CTLs express PD-1 in combination with other inhibitory receptors and exhibit symptoms of exhaustion (33, 35). Sustained PD-1 expression is linked to broad functional changes including reduced motility of activated CTLs during interactions with APCs (35). Whether PD-1 plays a role in maintenance of lymphoid T_RM_ cells has not been explored.

**Figure 3:**
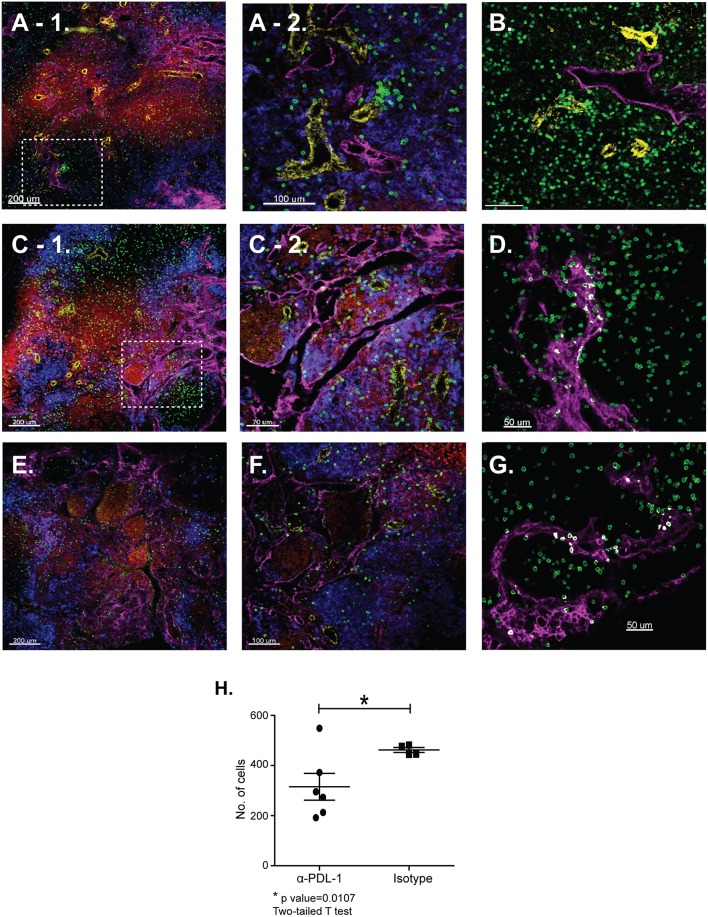
The distribution of lymphoid T_RM_ cells changes during PD-1L blockade. Naïve OTI cells were transferred to B6 mice 48 h before infection with WSN-OVA_I_. At 30 and 32 dpi, antibodies that block interactions with PD-L1 (or isotype control) were administered by IV injection (250 μg). After 35 days sections of fixed MLN were stained with antibodies that are specific for CD45.1 (green); CD31 (yellow); CD11c (blue); B220 (red), and LYVE-1 (magenta). Z-stacks were recorded at 10X and 20X normal magnification. **(A–D)** MLNs analyzed after treatment with control antibodies. **(E,F)** MLNs analyzed after treatment with antibodies that block interactions with PD-L1. The inset boxes (dashed lines) mark the locations of enlarged images shown in **(A-2,C-2)**. **(D,G)** The Imaris software colocalization function was used to detect contacts (white) between OTI cells (green) and LYVE^+^ vessels (Magenta). **(H)** Numbers of OTI cells per 10 micron Z-stack (*P = 0.0107).

**Figure 4:**
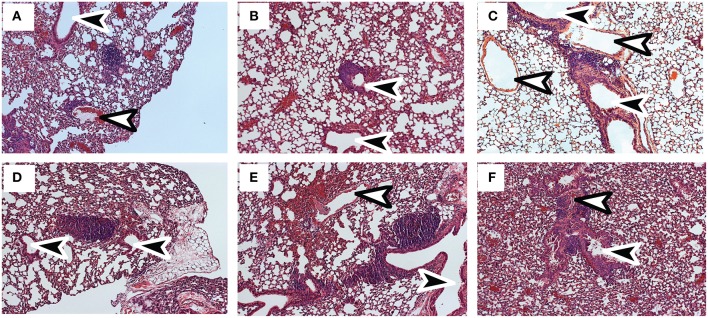
Infection-induced pathology is not substantially altered by PD-L1 blockade. C57BL/6 mice were infected with WSN-OVA_I_ and treated twice with **(A–C)** antibodies to PD-L1 or **(D–F)** isotype control. Sections of fixed lung tissue were stained 35 dpi using hematoxylin and eosin. Images were recorded at 20X normal magnification. Arrows indicate Bronchioles (black) and blood vessels (white).

Tetramer analyses showed that substantial numbers of anti-viral CTLs maintained PD-1 expression during recovery from IAV infection (Figure 1). To explore whether PD-1 plays a role in maintenance of lymphoid T_RM_ cells, we examined the distribution of antiviral CTLs in the MLNs of IAV infected mice during treatment with antibodies that block interactions with PD-1 ligand (PD-L1) (36).To visualize antiviral CTLs in the MLN, naïve (CD11a^low^CD44^low^) OTI cells were transferred to B6 mice 48 h before infection with WSN-OVA_I._ After 30 days, the recipient mice were treated twice with antibodies that block interactions with PD-L1, or an isotype control. MLNs were recovered 5 days after the first antibody treatment (35 dpi) and analyzed by scanning confocal microscopy (Figures 3A–G). OTI cells were widely distributed in the MLNs from both groups of animals. When control antibodies were used (Figures 3A–D) clusters of OTI cells (1 or 2 per MLN) were found in close proximity with high endothelial venules (HEVs) (Figure 3B), while additional OTI cells were adjacent to LYVE^+^ vessels. Areas of direct cell contact are shown in white (Figures 3D,G). No clusters of OTI cells were visible after PD-L1 blockade (Figures 3E–G). The numbers of CD45.1^+^ cells in the MLNs decreased during treatment with antibodies to PD-1L (Figure 3H). We found no difference in BrdU incorporation during the antibody treatments (data not shown). Taken together, these data indicate that small clusters of OTI cells dispersed during PD-L1 blockade, while some activated CTLs may have been released into the circulation. A single lobe from the lungs of each mouse was analyzed by H&E staining to evaluate changes in pathology during PD-L1 blockade (Figure 4). Infection-induced pathology was detected in the lungs of all animals and the areas of lymphocytic infiltration did not substantially change during treatment with antibodies that block PD-1L. Images were taken at 5X normal magnification and total areas of lymphocytic inflammation were measured using ImageJ software (*P* = 0.7221, *n* = 6/group).

### T_EFF_ Cells Enter the Lungs With Variable Kinetics After Heterosubtypic Challenge

**Figure 5:**
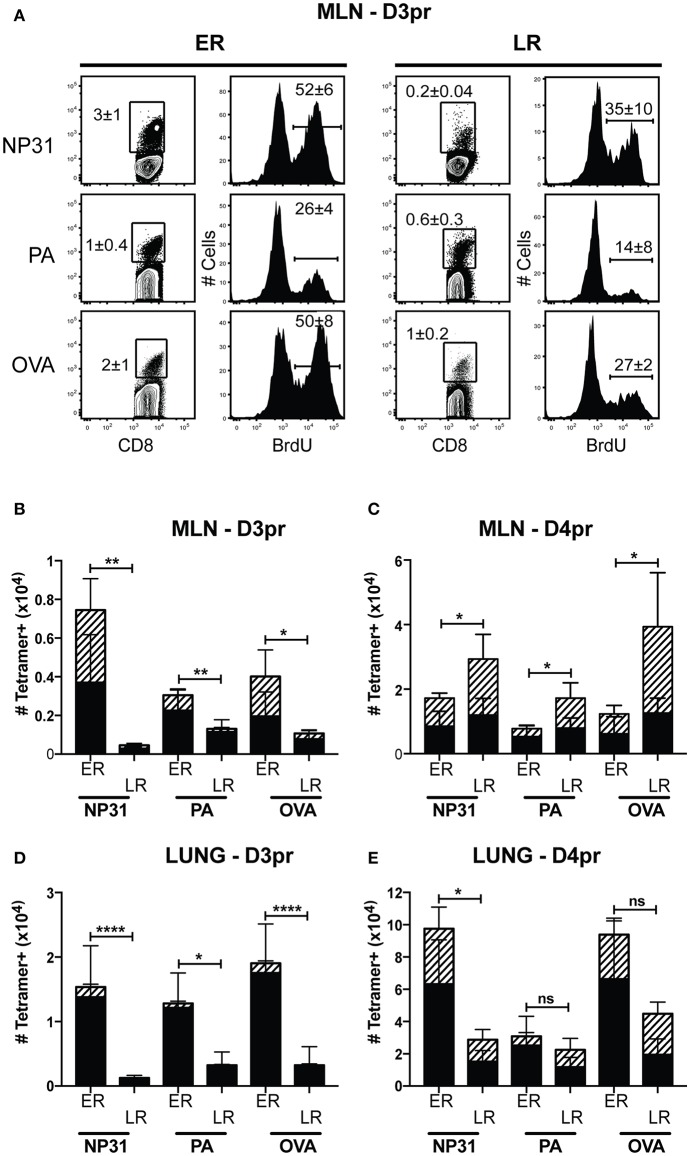
Early proliferation by lymphoid T_RM_ cells corresponds with increased numbers of anti-viral CTLs in the lungs. C57Bl/6 mice were infected with WSN-OVA_I_ and challenged with X31-OVA. Secondary infections were administered between 30 and 35 dpi (ER), or 120–160 dpi (LR). On the days indicated, each mouse received a single dose of BrdU (given by IP injection) and antiviral CTLs were analyzed 4 h later. **(A)** On day 3 post recall (D3pr), the MLNs were analyzed for antigen-experienced CTLs using high CD11a and CD44 expression. The contour plots show frequencies of tetramer^+^ cells. Histograms show BrdU incorporation within the tetramer gates. Percentages are means ± SD (*n* = 5/group). **(B–E)** The bar graphs show total numbers of Tetramer^+^ CTLs, including BrdU^+^ cells (hatched shading). Bars are means ± SD (*n* = 5/group). The numbers of cells that incorporated BrdU after ER and LR were compared using unpaired *T*-tests. NS, *P* > 0.05; *P < 0.05; **P < 0.01; ****P < 0.0001.

Heterosubtypic immunity disappears between 4 and 6 months after IAV infection (6, 37). To understand how lymphoid T_RM_ cells respond to secondary infection with a different strain of IAV, mice were primed with WSN-OVA_I_ (H1N1 serotype) and later challenged with X31-OVA (H3N2 serotype). Both viruses encode the SIINFEKL peptide, but express different surface proteins (25, 26). To explore how the functional properties of antiviral memory CD8 T cells evolve with time, we adjusted the interval between recurrent infections from 1 to 4 months. For optimal protection, secondary infections were administered between 30 and 35 dpi. For simplicity, this time interval is referred to as early recall (ER). To study the responses of antiviral memory CD8 T cells as immunity declined, additional mice were reinfected between 120 and 160 dpi. This interval is referred to as late recall (LR). On different days after secondary infection, groups of 5 mice received a single dose of BrdU and virus-specific CTLs were analyzed 4 h later (Figure 5). The contour plots show gated populations of antigen-experienced CD8 T cells (CD44-high, CD11a high) analyzed using tetramers specific for the NP, PA and OVA epitopes (Figure 5A). Unpaired student's *t*-tests were used to compare the rates of BrdU incorporation in the MLNs (3 dpi) during early and late recall. This experiment showed that anti-viral CTLs proliferated in MLNs with accelerated kinetics after early recall. The percentages of CD8 T cells that incorporated BrdU were significantly different for all three tetramers (NP, *P* = 0.0126; PA, *P* = 0.0205; and OVA, *P* = 0.0002). The bar graphs show total numbers of tetramer^+^ cells in the lungs and MLNs, with shading to indicate cells that contained BrdU (Figure 5B). Robust BrdU incorporation was not detected in the MLN until 4 days after late recall and T_EFF_ cells accumulated in the lungs with delayed kinetics. Weak proliferation by PA-specific CTLs contributed to a change in epitope dominance after reinfection, as reported previously (38, 39).

**Figure 6:**
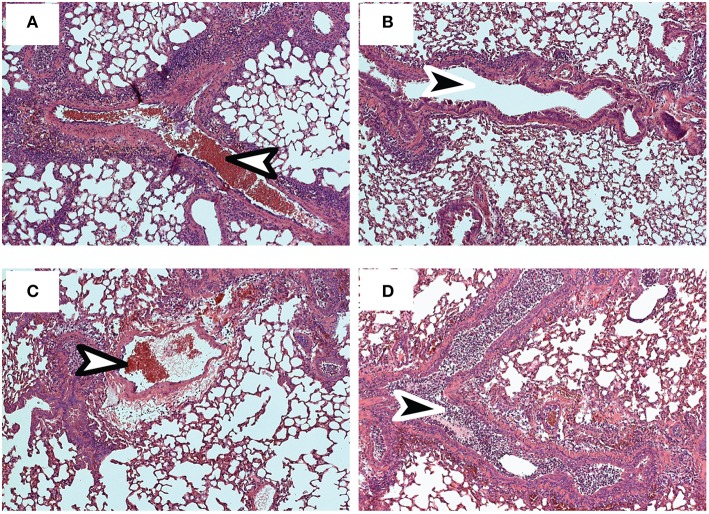
Sections of fixed lung tissue were stained with H&E 4 dpi. Representative images from 5 mice are shown. **(A,B)** Lungs after early recall **(C,D)** Lungs after late recall. Arrows indicate Bronchioles (black) and blood vessels (white).

Pathology in the lungs was evaluated 4 dpi, using H&E staining (Figures 6A–D). After early recall, the blood vessels were surrounded with pronounced mononuclear leukocytic infiltrates (Figure 6A), while the conducting airways were largely unobstructed (Figure 6B). Although perivascular and peribronchial infiltrates were less prominent after late recall (Figure 6C), the airways were heavily congested with mucus and mononuclear cells, including lung macrophages (Figure 6D). Taken together, these data show that lymphoid T_RM_ cells play an integral role in the response to reinfection and prompt dissemination of T_EFF_ cells to the lungs. As the interval between infections increased, lymphoid T_RM_ cells were replaced with T_CM_ cells which proliferated with delayed kinetics. We previously found that T_CM_ contributed to an exaggerated T_EFF_ response in the lungs as the infection progressed (6, 40), and does not prevent cellular obstruction in the airways.

## Discussion

Coordinated changes in homing receptor expression control the distribution of pathogen-specific CTLs in peripheral and lymphoid tissues. Naïve CD8 T cells follow gradients of sphingosine-1-phosphate (S1P) during transit through blood and lymph (41). Transit through lymphoid tissues is inhibited when CD8 T cells upregulate CD69 during antigen stimulation and the receptor for S1P (S1PR1) is modulated from the cell surface (42). A similar mechanism is required for T_RM_ cells to settle in infected tissues, where CD69 expression can be induced by local exposure to antigen and/or inflammation (43). Although the mechanism(s) that reinforce CD69 expression on T_RM_ cells have not been identified, chronic exposure to TGFβ prevents re-expression of S1P1 through negative regulation of Krupple-like factor 2 (KLF2) (23, 44). TGFβ also promotes retention of T_RM_ cells at barrier surfaces by maintaining CD103 expression, which in turn mediates interactions with a structural protein (E-cadherin) expressed on epithelial cells (17, 29, 45). Local concentrations of TGFβ increase during tissue repair and encourage T_RM_ cells to accumulate near inflamed tissues (46). Consistently, images of the lungs taken 30 dpi with IAV showed that the airway epithelium was densely populated with T_RM_ cells that expressed CD103 (6). Kinetic studies have shown that pulmonary T_RM_ cells gradually disappear from the lungs as protective immunity declines, while some replenishment occurs as small numbers of CTLs arrive from other tissues (15, 47). The origin of CTLs that enter the lungs during the recovery phase of infection is unknown, but may include CTLs that are released from the MLN during a response to persisting viral peptides (40).

Here, a two-step culture system has been used to define conditions that induce newly activated CTLs to express a canonical T_RM_ phenotype. We found that naïve OTI cells expressed CD69 in combination with CD103 soon after antigen stimulation. CD103 was eventually down regulated in the presence of antigen and returned during subsequent culture with TGFβ. A similar pattern was observed when naïve CD8 T cells were exposed to antigen *in vivo*. A transfer model also showed that naïve OTI cells completed multiple rounds of cell division in the MLN during late antigen presentation. Importantly, the responding CD8 T cells expressed CD103 when cultured with TGFβ. We previously showed that T_CM_ cells do not respond to late antigen presentation in the MLN 30dpi (16). When analyzed *in vitro*, T_CM_ cells did not express CD103 during culture with TGFβ, either before or after antigen stimulation. Together, these experiments show that naïve CD8 T cells respond to suboptimal antigen stimulation in the MLN and are receptive to factors in the local environment that promote extended residence in peripheral tissues. Importantly, CTLs become resistant to TGFβ after committing to the T_CM_ lineage.

Data from other models show that T_RM_ cells display variable phenotypes during infection with different pathogens. One report showed that T_CM_ cells trafficked to the skin during vaccinia virus infection and converted to CD69^+^ T_RM_ cells, without CD103 expression (48). Lymphoid T_RM_ cells displayed a similar phenotype during recovery from LCMV infection (19). To analyze CD8 T cells responses after IAV infection, we used mice that were bred in SPF facilities. Consequently, naïve CD8 T cells were the principal source of T_RM_ cells in this study. Tetramer analysis showed that the percentages of T_RM_ cells expressing CD103 varied according to antigen specificity (Figure 1). Whereas, large percentages of PA-specific T_RM_ cells expressed CD103, this marker was largely absent from NP-specific T_RM_ cells, while PD-1 was highly expressed in the lungs. It is important to note that the NP antigen is more abundant than PA during acute viral infection (49) and that NP peptides persist in the MLNs for approximately 2 months (15). Since CD8 T cells upregulate PD-1 and lose CD103 during antigen stimulation, many NP-specific T_RM_ cells displayed a phenotype (PD-1^+^CD69^+^CD103-negative) that was consistent with a response to persisting viral peptides (15). Some tumors contain intraepithelial lymphocytes (IEL) that express PD-1 in combination with CD103 (50). Some PA-specific T_RM_ cells expressed a similar phenotype in the lungs after IAV infection. Variations between these markers suggest that the phenotypes of T_RM_ cells are influenced by the timing and quantity or context of antigen exposure in local tissues.

We have found that T_CM_ cells respond to secondary IAV infection with delayed kinetics and contribute to a robust inflammation in the lungs as the infection progresses (6). The alveoli are surrounded by delicate membranes, which can be readily damaged by high concentrations of T cell-derived cytokines. Our data caution that some poorly designed vaccines may trigger a robust inflammatory response in the lungs during respiratory infection, by promoting formation of T_CM_ cells without T_RM_ in the local tissues (40, 51).

## Ethics Statement

Experiments were performed in accordance with guidelines and protocols approved by the University of Connecticut Health Center Institutional Animal Care and Use Committee (IACUC).

## Author Contributions

JS-R and KC were responsible for all experimentation and data processing. The Manuscript was written and edited by LC and JS-R. Histology was done by the histology core (UCONN Health) and evaluated by SB. All authors participated in editing the manuscript.

### Conflict of Interest Statement

The authors declare that the research was conducted in the absence of any commercial or financial relationships that could be construed as a potential conflict of interest.
